# Seasonal Expression of Glucose Transporter 5 (GLUT-5) Protein in the Testes of Roundleaf Bats in Thailand

**DOI:** 10.3390/ani15203003

**Published:** 2025-10-16

**Authors:** Saritvich Panyaboriban, Julaluk Jiangsakul, Navapol Kupthammasan, Baramee Chanchayanon, Apinya Poonnuan, Nidanis Hayeewaming, Nattamon Kumpasano, Sunate Karapan, Sayamon Srisuwatanasagul, Manita Wittayarat

**Affiliations:** 1 Faculty of Veterinary Science, Prince of Songkla University, Songkhla 90110, Thailand; saritvich.p@chula.ac.th (S.P.); julaluk.j@psu.ac.th (J.J.); navapol.k@psu.ac.th (N.K.); baramee.c@psu.ac.th (B.C.); apinya.poonnuan@gmail.com (A.P.); nidanis2@hotmail.com (N.H.); nhon0806@gmail.com (N.K.); 2 Department of Anatomy, Faculty of Veterinary Science, Chulalongkorn University, Bangkok 10330, Thailand; 3 Hala-Bala Wildlife Research Station, Hala-Bala Wildlife Sanctuary, Narathiwat 96160, Thailand; karapann@gmail.com

**Keywords:** sperm, fructose transporter, male bat, Hipposideridae, testicular biophysiology

## Abstract

**Simple Summary:**

Bats have unique reproductive strategies where sperm production and storage are aligned with seasonal conditions. This study examined how bats use fructose, an important energy source, by analyzing a sugar-transporting protein in the testes of three roundleaf bat species in Thailand during the breeding season. The protein was strongly expressed in developing sperm cells but absent in supporting cells, and its levels varied across months, reaching a peak in August during active spermatogenesis. Structural differences in the sperm-producing tissue were also found, with greater height and tubule size at the beginning of the season. Two forms of the protein were identified, each showing distinct patterns of expression, suggesting potential contributions to reproductive processes. These results highlight how the seasonal regulation of sugar transport and testicular architecture supports sperm maturation in bats. Such findings improve our understanding of reproductive biology in wild mammals and may offer insights into mechanisms of fertility regulation relevant to other species.

**Abstract:**

Bats have unique reproductive strategies that are closely related to testicular metabolic adaptations, such as prolonged sperm storage. This study examined the expression of glucose transporter 5 (GLUT-5), a fructose-specific member of the facilitative glucose transporter family, in the testes of roundleaf bats (*Hipposideros* spp.) collected from various locations in Thailand during their active reproductive season (July to September) and explored its association with biometric traits. To assess GLUT-5’s localization and expression levels, testicular tissues from 50 adult males representing *Hipposideros larvatus*, *Hipposideros armiger*, and *Hipposideros lekaguli* species were analyzed using immunohistochemistry and Western blotting. Strong GLUT-5 immunoreactivity was observed in the cytoplasm of spermatocytes, spermatids, and spermatozoa, while weak staining was seen in spermatogonia. No GLUT-5 expression was detected in Leydig or Sertoli cells. Staining intensity varied significantly by month, with the highest levels observed in August (*p* < 0.05), exceeding those in July and September. Western blotting identified two GLUT-5 isoforms (55 and 100 kDa), with relative intensities that changed across the reproductive timeline. In parallel, morphometric analysis revealed that the height of the germinal epithelium and the diameter of the seminiferous tubules were significantly greater in July than in August and September, reflecting peak spermatogenic activity. These findings suggest that the seasonal regulation of fructose transport, along with changes in testicular architecture, may support testicular function and sperm maturation. The differential expression of GLUT-5 isoforms may reflect their distinct roles in body growth, reproductive maturation, and seasonal testicular activity in Hipposiderid bats.

## 1. Introduction

Bats (order Chiroptera) account for one-fifth of all living mammals and comprise the second-largest order of the class Mammalia [1]. The Hipposideridae family, commonly known as Hipposiderid bats or roundleaf bats, is one of the largest bat families and is widely distributed across Thailand, comprising seven genera: *Anthops*, *Asellia*, *Aselliscus*, *Coelops*, *Doryrhina*, *Hipposideros*, and *Macronycteris* [2]. The mating system of bats is polygamous in multi-male/multi-female mating groups, and these bats undergo seasonal breeding. In tropical forests, male bats of the genus *Hipposideros*—which are seasonally monoestrous [3]—exhibit peak spermatogenesis in July, with reproductive organs changing in preparation for the breeding season in August [4,5]. In *H. larvatus*, testes are active from June to November, while ovulation occurs in December, and pregnancy takes place from January onwards [4]. Pregnant females of the *H*. *larvatus* (January–April) [4], *H. lekaguli* (March–April) [6], and *H. armiger* species have been found in the months from January to early May [7] or from April to June [8], depending on the study. This broadly overlapping reproductive timing allows data from these species to be collectively analyzed, providing a more comprehensive understanding of reproductive biology within the genus. Surprisingly, bat spermatozoa can prolong survival and storage in the female reproductive tract longer than that of other mammalian species, and they can survive in the oviduct of the female reproductive tract for several months [9]. One of the important mechanisms for this scheme is when sperm obtains a sufficient amount of nutrients for survival [10].

Mammalian spermatozoa can obtain energy through mitochondrial oxidative phosphorylation and glycolysis via monosaccharide consumption, especially fructose and glucose [11,12]. Fructose in semen can prevent premature sperm capacitation and maintain sperm metabolism at a level that promotes sperm survival [13]. GLUT-5 is a class II facilitative glucose transporter protein (GLUT), whose primary function is to mediate the uptake of fructose across the plasma membrane of living cells [14,15]. The fructose transportation in sperm cells is mediated through GLUT-5 for more than 90% of the time. Burant et al. [15] demonstrated that GLUT-5 expression could be a marker for the terminal maturity of male germ cells in humans. Moreover, it is associated with gluconeogenesis and glycogenesis, which sustain energy homeostasis and sperm survival in mammalian species. In the vespertilionid bat, the peak of testicular GLUT-5 expression was found during January, which corresponds to its mating period in Varanasi (25° N, 83° E), India. The increase in testicular GLUT-5 expression in bat male germ cells may be utilized to differentiate better-quality spermatozoa that can survive for a prolonged time in the bat female reproductive tract [10].

Given the seasonal reproduction of *Hipposideros* spp., this study aimed to investigate the spatial and temporal expression patterns of GLUT-5, a key fructose transporter, in the testes across reproductive stages. Immunohistochemistry and Western blotting were used to localize and quantify the 55 kDa and 100 kDa isoforms of the GLUT-5 protein. In addition to protein expression, morphometric data, including the height of the germinal epithelium, the diameter of the seminiferous tubules, and the area of interstitial tissue, were analyzed to assess testicular changes across months. By comparing samples collected across different months and locations, and correlating GLUT-5 expression with biometric traits, we sought to understand how variations in fructose transport may support testicular activity and reflect physiological or environmental adaptation in this species.

## 2. Materials and Methods

### 2.1. Sample Collection

In accordance with ethical guidelines for the use of wild mammals in research, male bat specimens were collected from several locations in Thailand, including the Wildlife Research Station Bueng Boraphet (15°40′18.85″ N, 100°14′13.84″ E), with samples from Sai Hai Sok Cave, Nang Kwak Cave, and Pradap Phet Cave; the Wildlife Research Station Chachoengsao (13°24′27.05″ N, 101°52′40.90″ E), where bats were obtained from Wat Khao Tham Rad, Khao Chakran Cave, and Khao Ang Rue Nai; and the Wildlife Research Station Khao Nang Ram (15°36′31.31″ N, 99°19′15.06″ E), with specimens collected from Prathun Cave, as shown in Table 1. Sample collection was carried out with permission from the Department of National Parks, Wildlife, and Plant Conservation. The specimen was deposited in the Mammal Collection of the Princess Maha Chakri Sirindhorn Natural History Museum, Prince of Songkla University (PSU), Hat Yai, Songkhla, Thailand (https://nhm.psu.ac.th/, accessed on 21 July 2022) under accession number PSUZC-MM.2021.6. Samples were obtained from a total of 50 male bats, including 39 *H. larvatus*, 4 *H. armiger*, and 7 *H. lekaguli*, collected from July to September 2019. The bats were weighed, and their forearm lengths were measured. Testes were dissected and cleaned, and tissue samples were taken from both testes of each individual, sectioned into small fragments (4 mm^3^, <20 mg), and preserved at −80 °C for immunoblotting. Additional samples were fixed in 4% (*w*/*v*) paraformaldehyde in PBS for immunohistochemical staining. All researchers complied with biosafety protocols for handling wild bats, including the use of protective gloves and masks, the administration of up-to-date rabies vaccinations, and the disinfection of all equipment after use during field collection. Similarly, museum-preserved specimens were handled under laboratory biosafety guidelines to ensure both researcher safety and sample integrity. The study protocol was submitted to the Animal Ethics Committee of Prince of Songkla University and was approved as an exempt protocol (Ref. AI149/2024).

### 2.2. Immunohistochemical Staining and Evaluation

**Figure 1:**
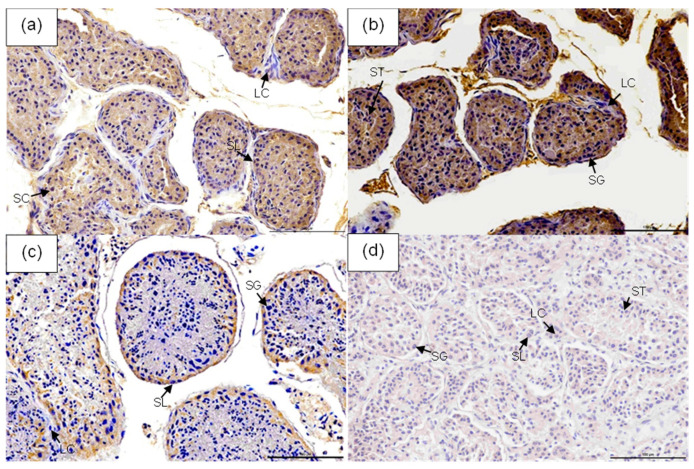
Immunohistochemical detection of GLUT-5 protein in the testes throughout July (**a**), August (**b**), and September (**c**). Negative control (**d**). Scale bars: 100 μm. LC = Leydig cell; SL = Sertoli cell; SG = Spermatogonium; SC = Spermatocyte; ST = Spermatid.

The fixed testes preserved in paraformaldehyde were embedded in paraffin blocks, sectioned at a thickness of 4 µm, and mounted onto coated slides. Immunohistochemistry (IHC) was performed with slight modifications to the protocol described by Wittayarat et al. [16]. Briefly, tissue sections were deparaffinized in xylene and rehydrated through a graded ethanol series. Endogenous peroxidase activity was blocked by immersing the sections in 3% (*v*/*v*) hydrogen peroxide in methanol at room temperature for 10 min. To reduce nonspecific staining, the sections were incubated with 10% normal goat serum (Abcam, Boston, MA, USA) for 1 h at room temperature, followed by overnight incubation at 4 °C in a moisture chamber with a rabbit polyclonal anti-GLUT-5 antibody (1:100, Abcam). The negative control was achieved using a matched IgG isotype control for rabbits at the corresponding concentration (1:100, Abcam; Figure 1d and Figure S1). The samples were rinsed three times in PBS for 5 min each, followed by incubation with a goat anti-rabbit IgG antibody conjugated to horseradish peroxidase (1:1000, Abcam) for 1 h in a moisture chamber at room temperature. Specific staining was then visualized using diaminobenzidine (DAB) (Abcam), and nuclei were counterstained with hematoxylin for 1–2 min. Finally, the sections were dehydrated and mounted with a glass coverslip.

The intensity of the immunostaining was subsequently semi-quantified in the DAB image. The images were obtained at 100× magnification under oil immersion utilizing a Nikon digital camera (Nikon Eclipse Ci-L; Nikon, Tokyo, Japan) with microscopic light intensity settings. The preserved images were examined utilizing Nikon NIS Elements (Basic Research (BR) version 5.21.00 Inc., Nikon Instruments, Tokyo, Japan) imaging software.

### 2.3. SDS-PAGE and Immunoblotting

SDS-PAGE and immunoblotting were performed with modifications to the protocol described by Wittayarat et al. [17]. All experiments were conducted in triplicate. Testis samples were minced and homogenized in radioimmunoprecipitation assay (RIPA) buffer containing a protease inhibitor (Complete-Mini; Roche-Boehringer, Ingelheim am Rhein, Germany). The lysates were then boiled in a Laemmli sample buffer supplemented with β-mercaptoethanol for 10 min. Equal protein loads (30 µg/25 µL) were loaded into each lane and separated by gel electrophoresis using a 10% acrylamide gel at 100 volts for 80 min. Proteins from all parts of the gel were transferred onto polyvinylidene fluoride (PVDF) membranes at 100 volts for 60 min using a wet transfer method. The membranes were then blocked in Tris-buffered saline (TBS) containing 10% (*w*/*v*) nonfat dry milk and 0.1% Tween 20 for 1 h at room temperature. Blots were incubated overnight at 4 °C with gentle shaking using primary antibodies against GLUT-5 (rabbit polyclonal antibody, 1:500, Abcam) and ß-actin (rabbit polyclonal antibody, 1:2000, Abcam). The blots were then incubated for 2 h at room temperature with horseradish peroxidase-conjugated goat anti-rabbit antibody (1:4000, Abcam). The negative control was achieved using a matched IgG isotype control for rabbits at the corresponding concentration. Because GLUT-5-positive control tissue in bats is technically difficult to obtain, antibody use in this study was supported by prior validation studies in the testis that were previously reported [18]. Samples from each month were analyzed to assess the relative levels of GLUT-5 protein compared to ß-actin from July to September.

Membranes were incubated with enhanced chemiluminescence (ECL) reagent (Cat. No. 170-5061, Bio-Rad Laboratories, Richmond, CA, USA). Protein bands were visualized using chemiluminescence (ECL; Thermo Scientific, Waltham, MA, USA) and developed with ImageQuant LAS 500 (GE Healthcare Biosciences AB, Uppsala, Sweden). Band intensities of ß-actin (42 kDa) and the target protein (GLUT-5, 55 and 100 kDa) were quantified using ImageQuant TL software (version 8.2.0.0, GE Healthcare Biosciences AB, Uppsala, Sweden). The mean intensity of protein bands for each month was recorded. For the semi-quantification of GLUT-5 protein intensities, the relative intensity levels of GLUT-5 at each molecular weight for each month were calculated using the following formula adapted from previous studies [19,20]:

$$\mathrm{Relative}\;\mathrm{intensity}\;\mathrm{of}\;\mathrm{GLUT}-5\;\left(55\;\mathrm{or}\;100\;\mathrm{kDa}\right)\;=\;\frac{\mathrm{Intensity}\;\mathrm{of}\;\mathrm{GLUT}-5\;(55\;\mathrm{or}\;100\;\mathrm{kDa})}{\mathrm{Intensity}\;\mathrm{of}\;ß-\mathrm{actin}\;(42\;\mathrm{kDa})}$$

### 2.4. Morphometric Data

Using the technique established by Srisuwatanasagul et al. [21], the testicular tissues were promptly dissected for morphometric examination following necropsy, subsequently preserved in 4% paraformaldehyde, minimally sectioned, and subjected to histological processing. All slides were subjected to staining with Hematoxylin and Eosin. The morphometric slides were captured with a digital slide scanner (3DHISTECH, Budapest, Hungary). Whole-slide digitalization was employed for each sample segment to visualize the complete image. Following slide scanning, testicular morphometrics were assessed using Caseviewer (version 2.4, 3DHISTECH, Budapest, Hungary). At least two hundred areas of seminiferous tubules were randomly selected from each slide, and the diameter of the seminiferous tubule and the height of the germinal epithelium were measured. The interstitial area was analyzed by assessing the interstitial area per mm^2^ of randomly selected testicular tissue.

### 2.5. Statistical Analyses

Statistical analyses were carried out using the Statistical Package for the R programming language (version 3.6.1) and R Studio (version 1.2.1335). All data, presented as the mean ± standard error of the mean (SEM), were compared for differences. The normality of the data was tested using the Shapiro–Wilk test. For immunohistological data, the intensity could not be normalized and was therefore analyzed using the Kruskal–Wallis test. Comparisons made with the Wilcoxon rank-sum test, adjusted for multiple tests, showed important differences in GLUT-5 intensity at different times or places. For the Western blot and morphometric data, the data were analyzed using a one-way ANOVA and then the Tukey test for post hoc analysis. Correlations for the total GLUT-5 relative intensity to body weight and forearm length, as well as for the relative intensity levels of GLUT-5 proteins (55 kDa) to GLUT-5 proteins (100 kDa), were determined using Pearson’s test for normal distribution or Spearman’s test for non-normal distribution. Results with *p* < 0.05 were considered statistically significant.

## 3. Results

### 3.1. Immunohistochemical Analysis of Seasonal GLUT-5 Expression in Testicular Tissue

Strong GLUT-5 immunoreactivity was observed in the cytoplasm of spermatocytes, spermatids, and spermatozoa, while weak staining was detected in spermatogonia. No GLUT-5 expression was detected in Leydig or Sertoli cells. As shown in Table 2 and Figure 1, the overall GLUT-5 protein intensity in the testes was significantly higher in samples collected in August (Figure 1b) compared to those from September (Figure 1c) and July (Figure 1a) (*p* < 0.05). This trend remained consistent when intensity values were normalized per mm^2^, indicating a greater localized abundance of GLUT-5 in testicular tissue during August. Negative control sections showed no staining (Figure 1d).

### 3.2. Spatial Variation in GLUT-5 Protein Expression in Bat Testicular Tissue Across Different Locations Revealed by Immunohistochemistry

Significant differences in GLUT-5 protein intensity were observed among the sampling locations (*p* < 0.05). The highest overall GLUT-5 expression was found in samples from Wat Khao Tham Rad (TR) and Khao Ang Rue Nai (AR), with intensity values significantly greater than those from Pradap Phet Cave (PP) (*p* < 0.05), which showed the lowest levels. When normalized to tissue area, Khao Chakran Cave (KC) exhibited the greatest GLUT-5 protein intensity per mm^2^, indicating a more concentrated localization of GLUT-5 in this region. In contrast, Pradap Phet Cave (PP) consistently demonstrated the lowest GLUT-5 intensity in both absolute and normalized measures. Detailed data are shown in Table 3.

### 3.3. Relative Expression Levels of GLUT-5 Proteins in Bat Testes

**Figure 2:**
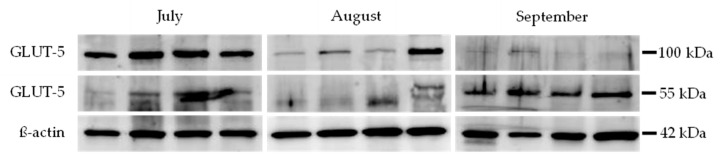
Representative Western immunoblot analysis of GLUT-5 (55 kDa and 100 kDa) and ß-actin (42 kDa) proteins in testis samples of *H. larvatus* throughout July, August, and September.

A Western immunoblot analysis revealed immunoreactive bands for GLUT-5 and β-actin proteins in representative samples, as shown in Figure 2. GLUT-5 was detected at molecular weights of 55 kDa and 100 kDa, while β-actin was observed at 42 kDa. Most testis samples exhibited both GLUT-5 isoforms, whereas some samples showed immunoreactivity at either 55 kDa or 100 kDa, independent of the month in which the sample was collected.

**Figure 3:**
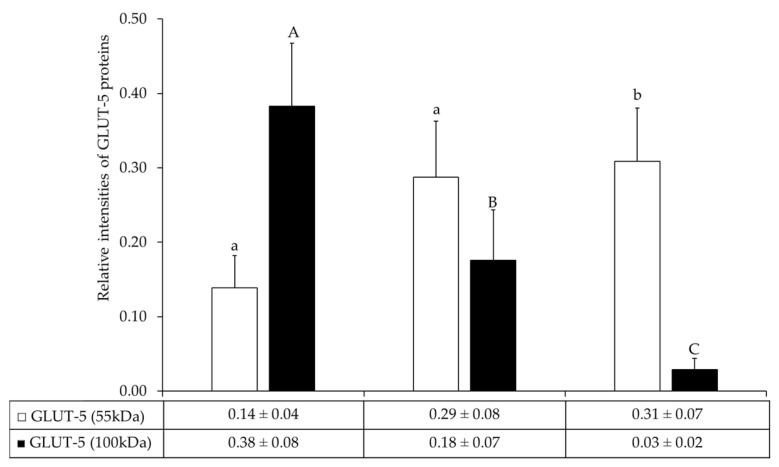
Graphs showing the presence (mean ± SEM) of relative GLUT-5 protein levels, comparing 55 kDa and 100 kDa in the testes of bats collected from July to September 2019. Different superscripts indicate significant differences in 55 kDa GLUT-5 (^a,b^, *p* < 0.05) and 100 kDa GLUT-5 between different months (^A–C^, *p* < 0.05).

The expression of GLUT-5 proteins varied across months. The relative intensity of GLUT-5 protein (55 kDa) was lowest in July (0.14 ± 0.04) and highest in September (0.31 ± 0.07) (Figure 3), while the expression of GLUT-5 (55 kDa) was significantly higher in testis samples collected in September compared to those collected in July (*p* < 0.05) (Figure 3). In contrast, the relative intensity of GLUT-5 protein (100 kDa) was highest in July (0.38 ± 0.08) and declined in August (0.18 ± 0.07) and September (0.03 ± 0.02), while GLUT-5 (100 kDa) expression was significantly higher in testis samples collected in July than in those collected in August and September (*p* < 0.05) (Figure 3).

### 3.4. Correlation of Body Weight and Forearm Length with GLUT-5 Protein Expression in Hipposiderid Bats

**Figure 4:**
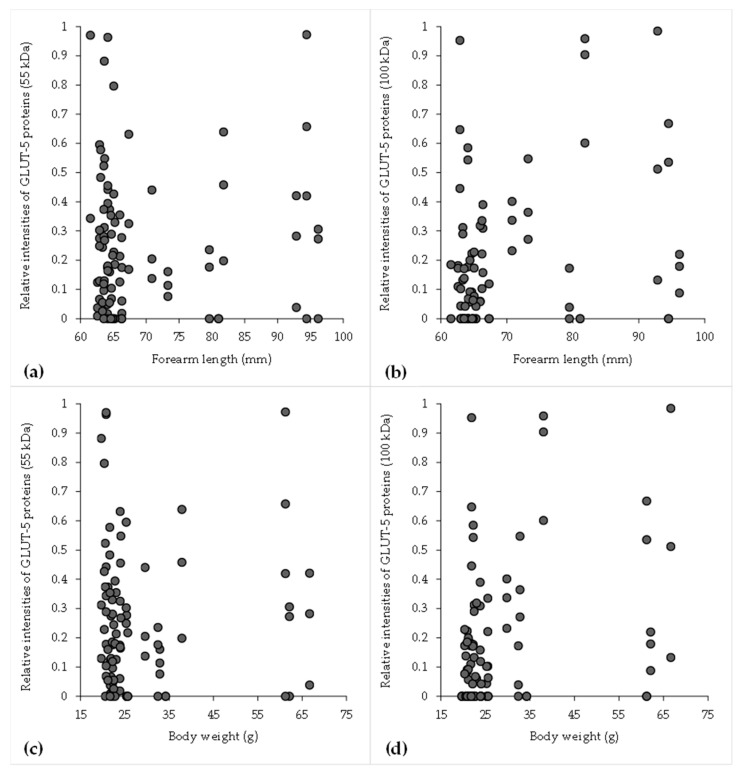
The scatter plot illustrates the correlation between GLUT-5 protein expression (55 kDa and 100 kDa), as determined by Western immunoblotting, and bat forearm length (mm) for 55 kDa (**a**) and 100 kDa (**b**), as well as body weight (g) for 55 kDa (**c**) and 100 kDa (**d**).

There was a high positive association between body weight and forearm length (n = 50), as indicated by the Pearson correlation coefficient of 0.968. Weight and forearm length were shown to be significantly correlated via linear regression analysis (*p* < 0.001), as shown in Table 4. The following regression equation was used: Length = 47.96 ± 0.757 × Weight. The relationship between the bat’s forearm length and body weight, as well as the relative intensity levels of total GLUT-5 proteins both in 55 kDa and 100 kDa, is shown in Figure 4. There was a weak positive association between the 55 kDa isoform of GLUT-5 protein intensity and both forearm length (r = 0.093, *p* = 0.128; Figure 4a) and body weight (r = 0.111, *p* = 0.069; Figure 4c), but there was an intermediate positive significant association between the 100 kDa isoform of GLUT-5 protein intensity and both forearm length (r = 0.262, *p* < 0.05; Figure 4b) and body weight (r = 0.272, *p* < 0.01; Figure 4d).

### 3.5. Morphometric Data

**Figure 5:**
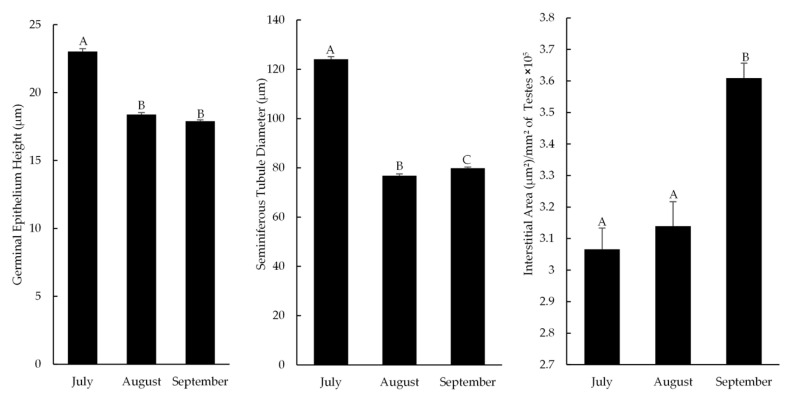
Graphs showing the presence of morphometric data (mean ± SEM) in bat testes at different collection times. Different superscript letters indicate significant differences (*p* < 0.05) between months.

As illustrated in Figure 5, the height of the germinal epithelium in the testicular tissue of roundleaf bats was significantly higher in July compared to August and September. Likewise, the diameter of the seminiferous tubules was significantly greater in July, but it increased in September compared to August. In contrast, the interstitial area of the testicular tissue was significantly larger in September than in both July and August.

## 4. Discussion

GLUT-5 is a facilitative glucose transporter protein involved in glucose and fructose uptake across cell membranes [22]. Previous mammalian studies reported GLUT-5 immunoreactivity mostly above 60 kDa, with a 65 kDa band detected in donkey sperm [23]. In the present study, two immunoreactive forms of GLUT-5 were found in the testes of *Hipposideros* species bats in Thailand. A major band appeared at 100 kDa, while a minor one appeared at 55 kDa. The 100 kDa form peaked in July, while the 55 kDa form was most abundant in September. This pattern aligns with the breeding season of *Hipposideros* spp., which begins in the latter part of the year, with ovulation occurring in December and pregnancy extending from January onwards into the first half of the year [4,6,7,8]. These findings correspond with those for *Scotophilus heathi*, where the 55 kDa GLUT-5 form is upregulated during the mating season (January to February) in India [10]. Together, these results suggest that the 55 kDa form may play a role in reproductive readiness in bats, although this remains a working hypothesis requiring further validation.

The 100 kDa GLUT-5 isoform, which has not been previously described, may represent either a precursor protein or a post-translationally modified mature protein. Proteolytic cleavage commonly activates precursor proteins [24], while modifications such as glycosylation, phosphorylation, or ubiquitination can alter molecular weight and electrophoretic mobility [25]. Glycosylation, in particular, is known to increase molecular weight by adding carbohydrate groups. Although specific modifications of GLUT-5 have been studied less than other GLUT family members, related transporters like GLUT-1 and GLUT-4 show that N-linked glycosylation clearly influences both molecular weight and cellular trafficking [26,27]. Given this, the 100 kDa band could reflect a glycosylated form of GLUT-5. Its peak in July may precede or contribute to the rise in the 55 kDa form in September, suggesting a seasonal shift in the testicular environment that supports sperm maturation and prepares them for storage in the female reproductive tract. This period coincided with a gradual decrease in ambient temperature from 28.9 °C in July to 27.8 °C in September (Thai Meteorological Department), which may also influence testicular physiology. Biochemical analyses such as enzymatic deglycosylation or mass spectrometry are needed to confirm these modifications [28].

The morphometric data of roundleaf bats in this study aligned with the findings of Srisuwatanasagul et al. [21], which showed that the height of the germinal epithelium and the diameter of the seminiferous tubules in *H. larvatus* peaked in July. This suggests that bats of this genus likely begin their breeding season in July, when ambient temperatures are relatively high. These parameters gradually declined as temperatures decreased from late August to September, supporting the conclusion that the active reproductive season in roundleaf bats occurs during the warmer months. In contrast, the interstitial area of the testicular tissue was significantly larger in September than in July and August, with histological analysis revealing thickening of the connective tissue between seminiferous tubules but without a corresponding increase in Leydig cell numbers.

The peak of spermatogenic activity in July corresponded with the highest expression of a 100 kDa isoform of GLUT-5, as revealed by Western blot analysis. This high-molecular-weight form, likely representing a mature glycosylated or oligomerized functional transporter, shows the strongest correlation with peak spermatogenic activity, with a sharp decline in expression in the following months. In contrast, the 55 kDa monomeric form does not correspond to the reproductive peak, and its accumulation later in the season may contribute to the total GLUT-5 signal detected by immunohistochemistry. Although immunohistochemical analysis showed the highest total GLUT-5 intensity in August, this is likely explained by the antibody detecting both isoforms or the combination of declining 100 kDa expression and accumulating 55 kDa protein as the breeding season came to an end. Overall, these findings indicate that the 100 kDa isoform of GLUT-5, rather than total GLUT-5 protein, serves as a more specific molecular marker for the peak metabolic and functional state of the testis, while acknowledging that biochemical confirmation (e.g., mass spectrometry or deglycosylation assays) is still required.

Morphological data revealed a strong positive correlation between body weight and forearm length (r = 0.968), which is consistent with established allometric relationships in bats [29]. These traits serve as useful indicators of physical maturity or nutritional status. In addition, this significant correlation suggests a high level of collinearity, indicating that both characteristics are accurate indicators of the same underlying factor: individuals’ general body sizes and physical conditions. Interestingly, the 55 kDa GLUT-5 isoform showed only a weak, statistically non-significant relationship with body size, while the 100 kDa form demonstrated a moderate, significant positive correlation with both body weight and forearm length. This suggests that 100 kDa GLUT-5 expression may be more closely linked to body growth, as reflected by body weight and forearm length. Since larger body size and longer forearms can indicate advanced developmental stages or better body condition, the greater intensity of this isoform may reflect metabolic adaptation or preparatory processes for reproductive activity. In contrast, the weak association of the 55 kDa form with body size may indicate a more baseline or constitutive role, suggesting that the 100 kDa isoform may be influenced by growth-related factors, hormonal activity, or seasonal metabolic changes. Further studies are needed to clarify its role in energy storage, nutrient transport, or reproductive physiology.

Regional variation in GLUT-5 expression also suggests that local environmental or physiological factors influence its testicular regulation. Elevated GLUT-5 levels in some populations may reflect heightened metabolic or reproductive activity, potentially tied to seasonal cycles or habitat quality. For instance, agricultural landscapes dominated by fruit orchards in Chachoengsao, where the Wildlife Research Station Chachoengsao is located, provide abundant fructose-rich resources that may enhance reproductive activity, whereas the limestone and dry forest habitats associated with the Wildlife Research Station Bueng Boraphet in Nakhon Sawan and the Wildlife Research Station Khao Nang Ram in Uthai Thani offer comparatively limited access to such diets. In contrast, consistently lower levels elsewhere may indicate delayed reproduction or variation in nutritional or hormonal cues. These findings underscore the importance of considering geographic differences in testicular metabolism and sugar transporter regulation. Although no direct studies have examined regional variation in GLUT-5 expression within bat populations, previous research in tropical species has shown that sugar transporter levels increase during reproductive peaks [30], which is consistent with mammalian models where fructose transporters are upregulated during spermatogenesis [31]. Ecological studies also indicate that higher-quality habitats support more intense reproductive activity [32], further supporting this interpretation.

Although seasonal changes in GLUT-5 expression are likely influenced by environmental cues such as the length of day, temperature, and food availability, our findings are based on samples collected during the reproductive season. Therefore, while the observed GLUT-5 levels may reflect heightened reproductive activity during the rainy season in Thailand (May–October), further studies with year-round sampling are needed to confirm seasonal patterns. Sperm storage in female bats extends the viable lifespan of sperm after mating until ovulation, which occurs months later [33]. GLUT-5 likely supports this strategy by facilitating sugar transport to sperm before ejaculation, ensuring their energy needs are met during prolonged storage. A similar function was reported by Roy and Krishna [10] for *Scotophilus heathi*, where GLUT-5 helped maintain sperm during prolonged storage within the female reproductive tract. Considering the close evolutionary relationship between *Hipposideros* spp. and members of the Rhinolophidae family, which are also known for sperm storage in females [1,34], it is likely that GLUT-5 contributes to a shared reproductive strategy. This strategy may involve increased expression of sugar transport proteins in the testes to support sperm survival over an extended period in the female tract. While the role of GLUT-5 in the male reproductive tract is increasingly understood, its function in the female reproductive tract remains underexplored. Future studies should investigate GLUT-5 expression in female tissues and the presence of alternative sugar sources such as glycogen near sperm storage sites [9,35,36]. Understanding these mechanisms will clarify how energy supply supports sperm viability during extended storage.

## 5. Conclusions

In conclusion, this study identified two GLUT-5 isoforms in Hipposiderid bat testes, with the 100 kDa form peaking earlier and the 55 kDa form peaking later in the reproductive season. Morphometric data showed that the height of the germinal epithelium and the diameter of the seminiferous tubules peaked in July, which may be associated with increased 100 kDa GLUT-5 expression. These findings indicate a possible involvement of GLUT-5 isoforms in structural alterations during seasonal sperm maturation. Regional differences in GLUT-5 expression may indicate environmental or physiological influences. Further studies, including additional protein validation, are needed to clarify the specific functions of each isoform.

## Figures and Tables

**Table 1:** Number of *H. larvatus*, *H. armiger*, and *H. lekaguli* individuals collected from different caves in Thailand during July–September.

Sampling Period/Place	*H. larvatus*	*H. armiger*	*H. lekaguli*
**July**	**10**	**1**	**3**
Sai Hai Sok Cave (SS)	3	-	-
Nang Kwak Cave (NK)	7	-	-
Pradap Phet Cave (PP)	-	1	3
**August**	**13**	**2**	**3**
Wat Khao Tham Rad (TR)	6	2	-
Khao Ang Rue Nai (AR)	4	-	-
Khao Chakran Cave (KC)	3	-	3
**September**	**16**	**1**	**1**
Prathun Cave (PT)	16	1	1
**Total**	**39**	**4**	**7**

**Table 2:** Comparison of GLUT-5 protein intensity in the testicular tissue of bats across three sampling months based on immunohistochemical analysis. Different letters in the same column indicate significant differences (*p* < 0.05).

Sampling Period	GLUT-5 Protein Intensity	GLUT-5 Protein Intensity per mm^2^
**July**	167.04 ± 1.51 ^a^	24.53 ± 1.02 ^a^
**August**	192.33 ± 1.54 ^c^	34.19 ± 0.94 ^b^
**September**	181.07 ± 1.65 ^b^	22.83 ± 0.92 ^a^

n = 50 males (39 *H. larvatus*, 4 *H. armiger*, and 7 *H. lekaguli*); July–September 2019.

**Table 3:** Variation in testicular GLUT-5 expression among sampling sites based on immunohistochemical findings. Different letters in the same column indicate significant differences (*p* < 0.05).

Location *	**GLUT-5 Protein Intensity**	**GLUT-5 Protein Intensity per mm^2^**
**SS**	170.40 ± 2.30 ^a^	25.98 ± 1.77 ^a^
**NK**	177.56 ± 1.92 ^b^	23.15 ± 1.29 ^b^
**PP**	145.97 ± 2.33 ^c^	16.82 ± 0.97 ^b^
**TR**	194.19 ± 2.95 ^d^	33.17 ± 1.27 ^c^
**KC**	185.22 ± 1.18 ^b^	43.56 ± 2.86 ^d^
**AR**	193.11 ± 1.00 ^d^	31.04 ± 1.21 ^c^
**PT**	175.22 ± 2.14 ^ab^	23.79 ± 1.10 ^ab^

* Abbreviations used for sampling locations: SS = Sai Hai Sok Cave; NK = Nang Kwak Cave; PP = Pradap Phet Cave (Wildlife Research Station Bueng Boraphet); TR = Wat Khao Tham Rad; KC = Khao Chakran Cave; AR = Khao Ang Rue Nai (Wildlife Research Station Chachoengsao); PT = Prathun Cave (Wildlife Research Station Khao Nang Ram).

**Table 4:** Correction analysis results predicting the relationship between forearm length, body weight, and GLUT-5 (55 kDa and 100 kDa) protein expressions in roundleaf bat testes.

	Forearm Length	Body Weight	GLUT-5	
			55 kDa	100 kDa
**Forearm length**	-	r = 0.968	r = 0.093	r = 0.262
		(*p* < 0.001)	(*p* = 0.128)	(*p* < 0.05)
**Body weight**	r = 0.968	-	r = 0.111	r = 0.272
	(*p* < 0.001)		(*p* = 0.069)	(*p* < 0.01)

## Data Availability

Data supporting the findings of this study are available from the corresponding author upon reasonable request.

## Supplementary Materials

The following supporting information can be downloaded at: https://www.mdpi.com/article/10.3390/ani15203003/s1, Figure S1: Isotype control for immunohistochemical staining of testis section.
