# Supplementary material for: Seasonal Expression of Glucose Transporter 5 (GLUT-5) Protein in the Testes of Roundleaf Bats in Thailand
Source: Animals (Basel). 2025 Oct 16;15(20):3003. doi: 10.3390/ani15203003 (PMC12560875; doi:10.3390/ani15203003)
Supplement: Supplementary file 1 [file animals-15-03003-s001.zip › animals-3898862-supplementary.pdf]

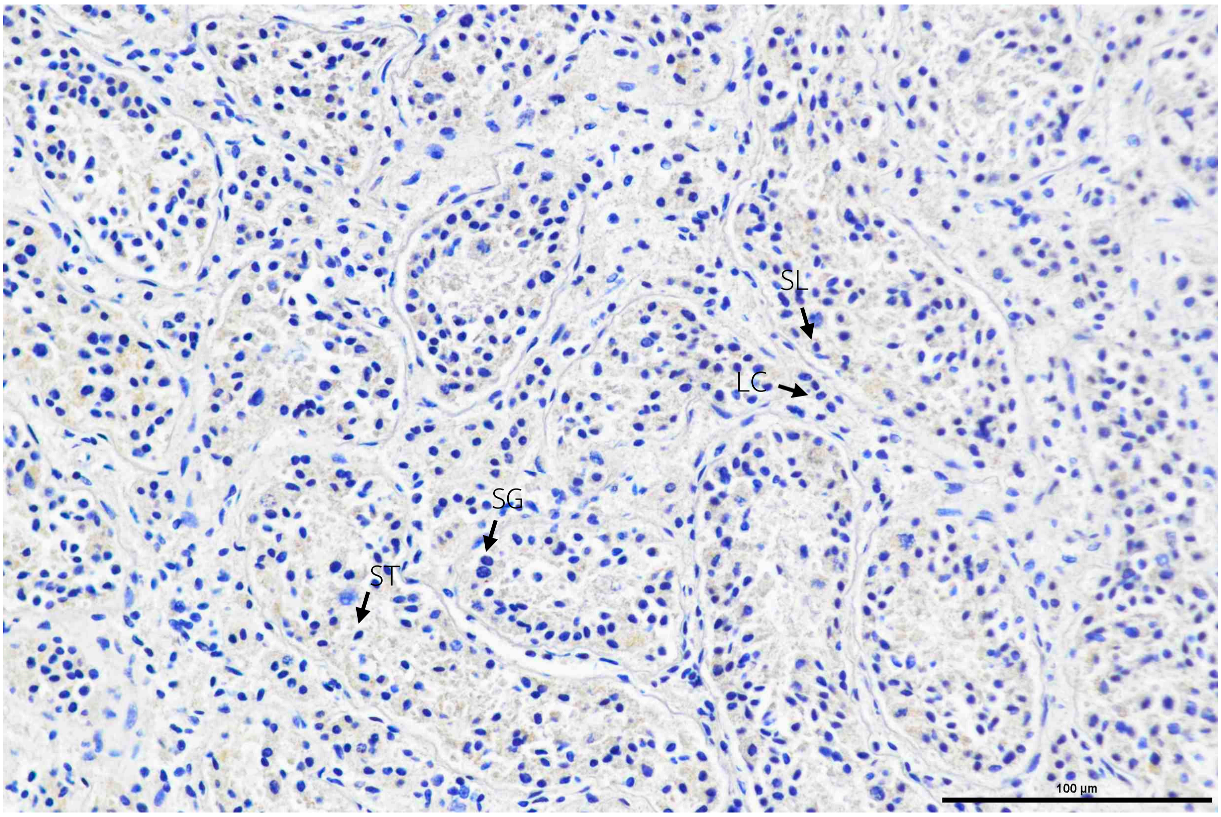

Figure S1. Representative image of immunohistochemical staining of a testis section using a rabbit IgG isotype control at the corresponding concentration (1:100; negative control). No specific staining was observed. Scale bar: 100 µm. LC = Leydig cell; SL = Sertoli cell; SG = Spermatogonium; ST = Spermatid.
